# Two-Stage Hepatectomy for Cholangiocarcinoma Under Total Vascular Exclusion with Veno-venous ECMO Bypass and Controlled Closed-loop In Situ Hypothermic Oxygenated Perfusion of the Liver: The Hannover CLIP-Concept

**DOI:** 10.1245/s10434-025-18346-x

**Published:** 2025-10-28

**Authors:** Cornelius J. van Beekum, Philipp Felgendreff, Simon Störzer, Nora Nevermann, Hendrik Eismann, Christian Kühn, Thomas Wirth, Anna Saborowski, Björn Hartleben, Judith Pantke, Markus Quante, Tung Yu Tsui, Moritz Schmelzle

**Affiliations:** 1 Department of General, Visceral and Transplant Surgery, Hannover Medical School, Hannover, Germany; 2 Department of Anaesthesiology and Intensive Care Medicine, Hannover Medical School, Hannover, Germany; 3 Department of Gastroenterology, Hepatology, Infectious Diseases and Endocrinology, Hannover Medical School, Hannover, Germany; 4 Institute for Pathology, Hannover Medical School, Hannover, Germany; 5 Department for Cardiac, Thoracic, Transplantation and Vascular Surgery, Hannover Medical School, Hannover, Germany; 6 Department of Diagnostic and Interventional Radiology, Hannover Medical School, Hannover, Germany

## Abstract

**Background:**

Total vascular exclusion (TVE) enables the resection of centrally located liver tumors but remains associated with considerable intra- and perioperative morbidity. We present the Hannover Modification of TVE, which combines veno-venous extracorporeal membrane oxygenation (vvECMO) and a closed-loop in situ hypothermic oxygenated perfusion (CLIP) of the liver using the Bridge-to-Life^®^ VitaSmart system for targeted parenchymal protection.

**Methods:**

A 62-year-old woman with FGFR2-fused intrahepatic cholangiocarcinoma (iCCA) involving the hepatocaval confluence and all three hepatic veins, previously deemed unresectable, underwent partial ALPPS-preserving segment IVb. One week later, extended right trisectionectomy with reconstruction of the left hepatic vein was performed under TVE. Dual perfusion circuits were established: (1) a portocaval anastomosis was established and systemic and portal venous return was maintained via vvECMO; (2) cold (4 °C), oxygenated HTK solution was infused via a left portal vein catheter, drained through the hepatic veins into the IVC, and recirculated through a caval outflow cannula.

**Results:**

The CLIP approach ensured continuous oxygenation and hypothermia of the liver during resection and venous reconstruction without systemic cooling. Operative time was 4 hours and 3 minutes, with 72 minutes of CLIP and 130 minutes of vvECMO. Histopathology revealed a 6.5-cm iCCA (ypT1a, G2) with negative margins (R0). The postoperative course was uneventful, and the patient was discharged on postoperative day 7 with excellent liver function.

**Conclusions:**

The Hannover CLIP technique effectively combines controlled, recirculated HOPE with vvECMO. This approach minimizes ischemic injury to the liver, kidneys, and intestines and facilitates safe resection of highly complex central liver tumors under TVE.

**Supplementary Information:**

The online version contains supplementary material available at 10.1245/s10434-025-18346-x.

## Background

Total vascular exclusion (TVE) of the liver is a surgical technique employed for the resection of centrally located liver tumors involving the inferior vena cava (IVC) or the hepatocaval confluence (HC). Total vascular exclusion involves supra- and infrahepatic clamping of the IVC, combined with occlusion of portal venous and arterial inflow. When used in conjunction with in situ cold perfusion using organ preservation solution, TVE facilitates a bloodless surgical field optimized for safe and radical parenchymal dissection, as first described by Fortner et al., and further developed by Rudolf Pichlmayr.^1,2^

Despite significant technical progress, TVE continues to induce substantial ischemia-reperfusion injury, contributing to considerable morbidity and mortality.^3,4^ To mitigate these effects, adjunctive strategies such as veno-venous bypass and hepatic hypothermia—adopted from liver transplantation—have been implemented. Conventional cooling typically involves a single flush of the liver with cold preservation solution, which drains freely into the abdominal cavity. This uncontrolled drainage may result in systemic hypothermia and fluid absorption, leading to electrolyte disturbances and coagulopathy.

Recent advances in transplant technology have introduced devices for continuous, oxygenated hypothermic perfusion, aiming to reduce ischemia-reperfusion injury. In a recent case, Tribolet et al. reported the use of the Bridge-to-Life^®^ device for in situ hypothermic portal perfusion during a left hepatectomy under TVE.^5^ While this approach demonstrated effective reduction in hepatic ischemia, it did not address the uncontrolled efflux of perfusate into the operative field.

**Fig. 1:**
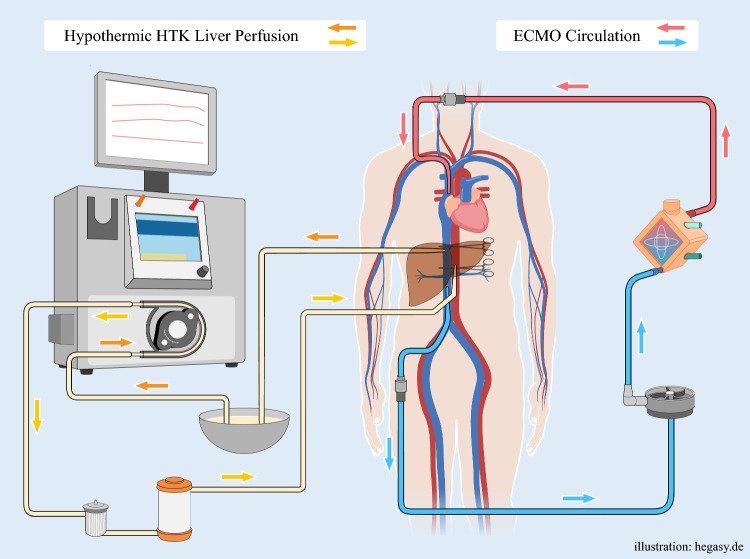
Perfusion set up of closed-loop in situ perfusion (CLIP)

The Hannover Modification introduces a novel concept of closed-loop in situ liver perfusion (CLIP), in which organ preservation solution is recycled through IVC cannulation and recirculated via a perfusion system (Fig. 1). This technique integrates transplant-derived strategies into complex oncologic liver resections, enabling controlled hypothermic oxygenated perfusion of the liver remnant while avoiding systemic hypothermia and minimizing contamination of the peritoneal cavity with preservation solution.

Here, we present our initial clinical experience with the CLIP technique, performed under TVE and combined with veno-venous extracorporeal membrane oxygenation (vvECMO).

## Patient and Methods

A 62-year-old woman with FGFR2-fused intrahepatic cholangiocarcinoma mainly located in segments I, IVa, VIII with involvement of all three hepatic veins and the HC, was initially deemed unresectable at another center. She received platinum-based chemotherapy and immune checkpoint inhibition. Following partial response, resection was attempted but aborted at a different center due to persistent venous involvement. Given the presence of an FGFR2-fusion, the patient was continued on systemic therapy with Lirafugratinib, a highly selective FGFR2 inhibitor. After 2 years of systemic therapy, restaging revealed stable disease without extrahepatic spread, and the patient was referred to Hannover Medical School. Given the favorable tumor biology and excellent general health, curative resection was reconsidered.

### Surgical Technique

**Fig. 2:**
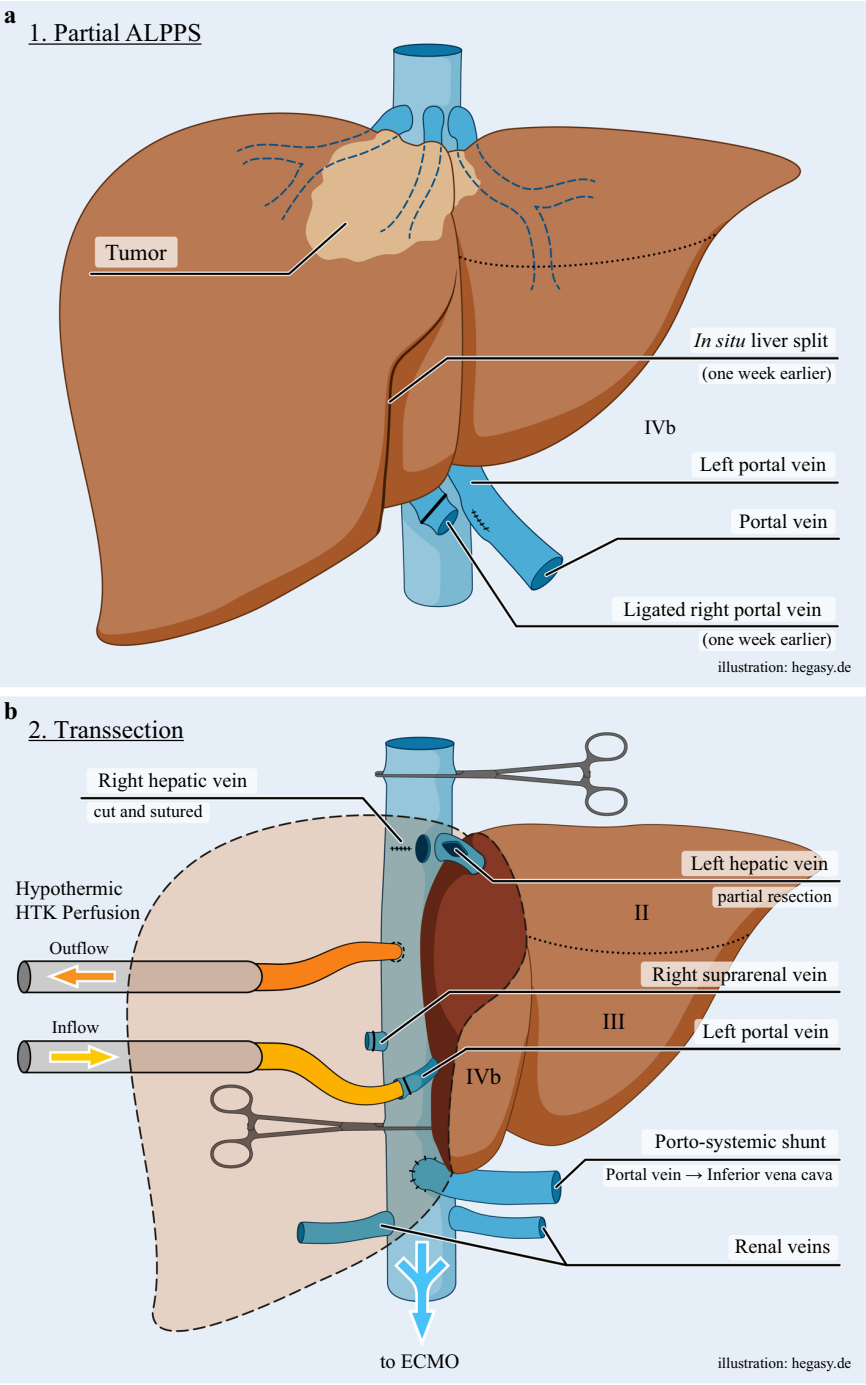

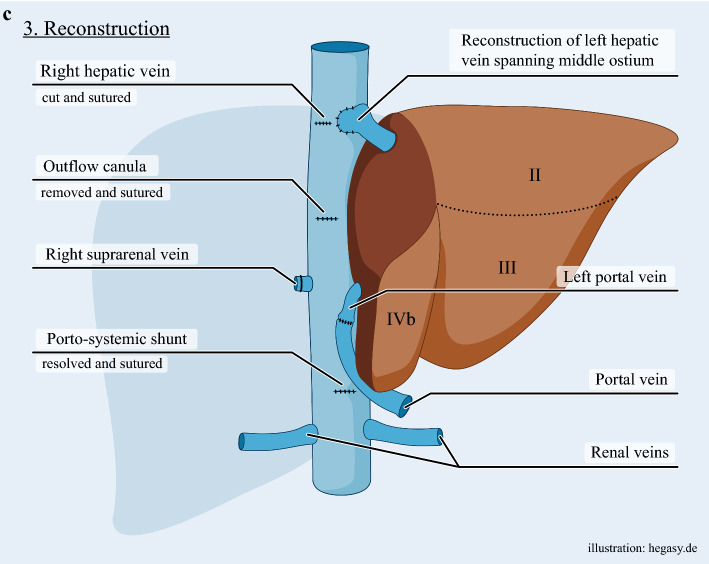
**a** Partial ALPPS procedure for liver augmentation prior to trisectionectomy. **b** Cannula placement and resection plane.**c** Reconstruction of the portal vein and hepatocaval confluence

Hypertrophy of liver segments II, III, and IVb was induced by partial associating liver partition with portal vein ligation for staged hepatectomy (partial ALPPS; Fig. 2a). The caudal parenchymal dissection line started between segments IVb and V and switched to the left between the left lateral and left medial segments aiming to preserve segments II, III, and IVb. On postoperative day 7, CT confirmed adequate hypertrophy of the future liver remnant. Final resection proceeded on day eight.

To prevent intestinal and renal venous congestion and to protect the liver from warm ischemia, a multidisciplinary strategy combining total vascular exclusion, vvECMO, and hypothermic oxygenated perfusion was developed. Extracorporeal membrane oxygenation (ECMO) was established via femoral (outflow) and jugular (inflow) cannulation. Simultaneously, the Bridge-to-Life^®^ VitaSmart system delivered 4 °C oxygenated preservation solution to the liver remnant via a left portal vein cannula. Veno-venous ECMO was chosen over a standard veno-venous bypass for a stable and reliable extracorporeal circuit with integrated temperature and flow control, which we deemed essential in this extended resection under total vascular exclusion. Owing to the anticipated duration of vascular exclusion and the complexity of dual perfusion, ECMO offered enhanced hemodynamic stability and safety, especially with regard to intestinal and renal venous drainage without the need for additional roller pumps or manual interventions.

Relaparotomy was performed, the right adrenal vein was transected, and systemic heparin (5,000 IU) administered before clamping. The IVC was clamped above the renal veins and below the hepatic veins. A drainage cannula was inserted into the retrohepatic vena cava between the clamps. The right hepatic artery was ligated, and the common bile duct and portal vein were encircled. The portal vein and its left branch were transected; a portosystemic shunt was established via end-to-side anastomosis of the portal vein with the clamped IVC, thereby allowing intestinal venous decompression and avoiding dual cannulation of the portal vein/inferior mesenteric vein (Fig. 2b).

The caudal IVC clamp was repositioned between the caval outflow catheter and portocaval shunt; the superior caval clamp was moved cranially above the hepatic veins thereby completely excluding the liver (TVE) and allowing portal venous blood to drain from the infrahepatic cava into the ECMO. A CLIP-cannula was inserted into the left portal vein and continuous oxygenated perfusion was initiated. The perfusate drained via hepatic veins into the IVC, was collected via the caval cannula, reoxygenated, and recirculated to the portal system, thereby maintaining hypothermia and oxygenation of the liver remnant without systemic cooling.

Parenchymal transection included en-bloc resection of segments I, IVa, V, VI, VII, and VIII. The left hepatic artery and bile duct were preserved. The right and middle hepatic veins were divided at the HC. Owing to tumor infiltration, the cranial medial wall of the left hepatic vein was excised and the remaining lateral wall was reconstructed in a diamond-shaped anastomosis to the united middle and left hepatic vein ostium (Fig. 2c).

The portosystemic shunt was removed, and portal continuity restored via end-to-end portal vein anastomosis (Fig. 2c). Four hundred milliliters of warm blood were flushed through the remnant. Both IVC clamps and all cannulas were removed, ECMO discontinued.

## Results

Total operative time was 4 hours and 3 minutes. ECMO duration was 130 minutes; perfusion lasted 72 minutes with 8 liters of HTK solution at 4 °C. Owing to ECMO-related blood loss and hemodilution, the patient received 7 units of packed red blood cells and 8 units of fresh frozen plasma intraoperatively.

Histopathological examination showed a 6.5 cm fibrotic mass with ~10% viable, moderately differentiated adenocarcinoma (G2), consistent with treated cholangiocarcinoma. Resection was complete (R0) without vascular or perineural invasion: UICC (8th ed.): ypT1a (1.5 cm), pNx, L0, V0, Pn0, G2, R0, M0.

### Postoperative Course

After postoperative observation on the intensive care unit, the patient was transferred to the surgical ward on postoperative day 2. Drains were removed on day 5 with no evidence of biliary leakage. A right-sided pleural effusion was managed conservatively (Clavien-Dindo II). The patient was discharged in good condition and with normal liver function on day 7 after the second operation.

## Discussion

This case illustrates the integration of modern transplant-based strategies in complex oncologic liver resection. The patient's FGFR2-fused tumor responded favorably to lirafugratinib, allowing for delayed but successful resection following systemic therapy.

While TVE with in situ hypothermic perfusion has been described previously, conventional techniques often result in intraoperative hypothermia with the associated complications. The Hannover CLIP modification combines closed-loop in situ hypothermic oxygenated perfusion of the liver and extracorporeal venous bypass, thereby minimizing intestinal and renal venostasis, preventing systemic cooling, and facilitating oncologic radicality under safe vascular exclusion.

In this case, two independent perfusion circuits were established under TVE: (1) vvECMO for hemodynamic stability and venous return from the kidneys and intestines; and (2) a closed-loop perfusion circuit via the left portal vein to the liver remnant, with venous outflow via hepatic veins into the IVC and recirculation through a sterile reservoir. This strategy maintained consistent, exclusive liver hypothermia and oxygenation and enabled bloodless resection of a central cholangiocarcinoma with reconstruction of the portal vein and left hepatic vein.

In contrast to the in situ hypothermic oxygenated portal perfusion described by Boubaddi et al., which utilized a nonrecirculating system with uncontrolled efflux of perfusate into the operative field, and the ante situm or in situ perfusion techniques of Cillio and Azoulay, which require complete hepatic mobilization and ex vivo handling, the Hannover CLIP concept maintains the liver in situ and establishes a fully closed, continuously recirculating and oxygenated perfusion circuit.^5–7^

To our knowledge, this modification represents the first reported application of combined TVE, vvECMO, and closed-loop in situ hepatic perfusion using the Bridge-to-Life VitaSmart system in liver surgery.

## Supplementary Information

Below is the link to the electronic supplementary material.

Supplementary file1 (MP4 979200 KB)

## Data Availability

The data are available upon reasonable request from the authors.
